# The Chaperonin TRiC/CCT Inhibitor HSF1A Protects Cells from Intoxication with Pertussis Toxin

**DOI:** 10.3390/toxins16010036

**Published:** 2024-01-10

**Authors:** Jinfang Jia, Manuel Zoeschg, Holger Barth, Arto T. Pulliainen, Katharina Ernst

**Affiliations:** 1 Institute of Experimental and Clinical Pharmacology, Toxicology and Pharmacology of Natural Products, Ulm University Medical Center, 89081 Ulm, Germany; 2 Institute of Biomedicine, University of Turku, FI-20520 Turku, Finland

**Keywords:** pertussis toxin, TRiC/CCT, chaperonin, cellular uptake, toxin inhibitor, AB-type toxin

## Abstract

Pertussis toxin (PT) is a bacterial AB_5_-toxin produced by *Bordetella pertussis* and a major molecular determinant of pertussis, also known as whooping cough, a highly contagious respiratory disease. In this study, we investigate the protective effects of the chaperonin TRiC/CCT inhibitor, HSF1A, against PT-induced cell intoxication. TRiC/CCT is a chaperonin complex that facilitates the correct folding of proteins, preventing misfolding and aggregation, and maintaining cellular protein homeostasis. Previous research has demonstrated the significance of TRiC/CCT in the functionality of the *Clostridioides difficile* TcdB AB-toxin. Our findings reveal that HSF1A effectively reduces the levels of ADP-ribosylated Gαi, the specific substrate of PT, in PT-treated cells, without interfering with enzyme activity in vitro or the cellular binding of PT. Additionally, our study uncovers a novel interaction between PTS1 and the chaperonin complex subunit CCT5, which correlates with reduced PTS1 signaling in cells upon HSF1A treatment. Importantly, HSF1A mitigates the adverse effects of PT on cAMP signaling in cellular systems. These results provide valuable insights into the mechanisms of PT uptake and suggest a promising starting point for the development of innovative therapeutic strategies to counteract pertussis toxin-mediated pathogenicity.

## 1. Introduction

Pertussis toxin (PT) of *Bordetella (B.) pertussis* plays an important role in causing the disease pertussis, also known as whooping cough, which is characterized by distinct stages of symptoms [1,2,3,4]. The initial stage resembles a cold with a runny nose, mild cough, and low-grade fever. The paroxysmal stage follows, characterized by severe coughing fits with a “whooping” sound, sometimes leading to vomiting and exhaustion. The final stage involves gradual improvement, but the cough may persist for weeks. While vaccination can result in milder symptoms, whooping cough can be severe, especially in infants, and may lead to complications like pneumonia and seizures. Early diagnosis and medical attention are crucial [3,5,6,7].

According to a report from the World Health Organization (WHO) in 2014, pertussis had a significant impact on young children with more than 24.1 million cases and 160,700 deaths reported globally [8,9]. Despite widespread vaccination efforts, particularly in Western countries, the incidence of pertussis has been on the rise, reaching levels not seen since the vaccine’s introduction in the 1950s [9,10,11,12,13].

While antibiotics can effectively eliminate *B. pertussis* bacteria, they do not provide relief from pertussis symptoms if administered after the first two weeks of infection, which is often due to delayed diagnoses [3,14,15,16]. Studies have shown that PT is responsible for causing prolonged and severe inflammation in the airways, as demonstrated in mouse models [17]. Notably, *B. pertussis* strains lacking PT expression do not lead to severe symptoms like leukocytosis or death, emphasizing the critical role of PT, especially in severe cases [1,18,19]. Consequently, PT has become a focal point for developing innovative therapeutic approaches [20].

The limited treatment options for whooping cough highlight the necessity to explore new PT-targeted inhibitors as potential treatments. This research is crucial for combating the ongoing challenges posed by pertussis and improving outcomes for affected individuals.

PT is classified as an AB_5_ toxin, characterized by a distinctive structure consisting of an enzyme subunit, referred to as A-protomer PTS1, and five B-subunits forming a pyramid-shaped holotoxin [21,22,23,24]. Once released by *B. pertussis*, the B-pentamer of PT interacts with sialoglycoproteins found on different cell types, facilitating its binding to the cell surface [25,26,27]. The toxin is then internalized through endocytosis and transported retrogradely within the cell, moving from the Golgi to the endoplasmic reticulum (ER) [28,29].

At the ER, the PTS1 subunit dissociates from the B-pentamer in an ATP-dependent manner [30,31,32]. Under physiological conditions, the PTS1 is thermally unstable and undergoes unfolding [33,34]. Consequently, the ER-associated degradation (ERAD) pathway recognizes the unfolded PTS1 and transports it from the ER to the cytosol for further processing. Importantly, the lack of lysine residues in PTS1 prevents ubiquitination and subsequent proteasomal degradation [35,36]. 

Once inside the cytosol, PTS1 engages in an enzymatic process known as ADP-ribosylation. It transfers an ADP–ribose group from its co-substrate NAD^+^ onto the α-subunits of inhibitory G-proteins (Gαi) [37,38,39]. As a consequence of this cysteine-specific C-terminal ADP-ribosylation the Gαi subunits lose their ability to interact with G-protein coupled receptors (GPCRs) and thereby cannot function as negative regulators of adenylate cyclase (AC). This disruption in the normal functioning of Gαi leads to disturbances in cAMP signaling within the cell. 

TRiC/CCT functions as a molecular machinery responsible for the correct folding of numerous recently synthesized eukaryotic proteins [40,41]. It appears that around 10% of cytosolic proteins engage with TRiC/CCT [40,42]. The role of the chaperonin TRiC/CCT is crucial in ensuring the proper folding of various cytoskeleton proteins, such as actin [43,44,45] and tubulin [42,46]. It was recently shown that the AB-type toxins of *Clostridioides (C.) difficile*, TcdA and TcdB, require the assistance of the chaperonin TRiC/CCT to restore their activity after translocating into the target cell cytosol [47]. This study used the cell-permeable small molecular weight inhibitor of TRiC/CCT, HSF1A [47,48]. It was shown that HSF1A directly interacts with TRiC/CCT, reducing its thermal stability and impairing the TRiC/CCT-mediated folding of actin while leaving ATP hydrolysis unaffected [48,49]. Additionally, the inhibition of TRiC/CCT by HSF1A triggers the activation of heat shock transcription factor 1 (HSF1). Consequently, this activation prompts the expression of genes responsible for safeguarding proteins against stress, including those encoding proteins crucial for protein folding, such as Hsp70 [48]. Since it is known that PT requires the activity of other chaperones, namely Hsp90, Hsp70, cyclophilins (Cyps), and FK506 binding proteins (FKBPs) [20,50,51,52,53,54], for uptake and activity of PTS1 in the cytosol, the inhibitory potential of HSF1A on PT was investigated in this study.

## 2. Results

### 2.1. In PT-Treated Cells, HSF1A Reduces the Levels of ADP-Ribosylated Gαi without Causing Any Interference with the Enzyme Activity In Vitro or the Cellular Binding of PT

**Figure 1:**
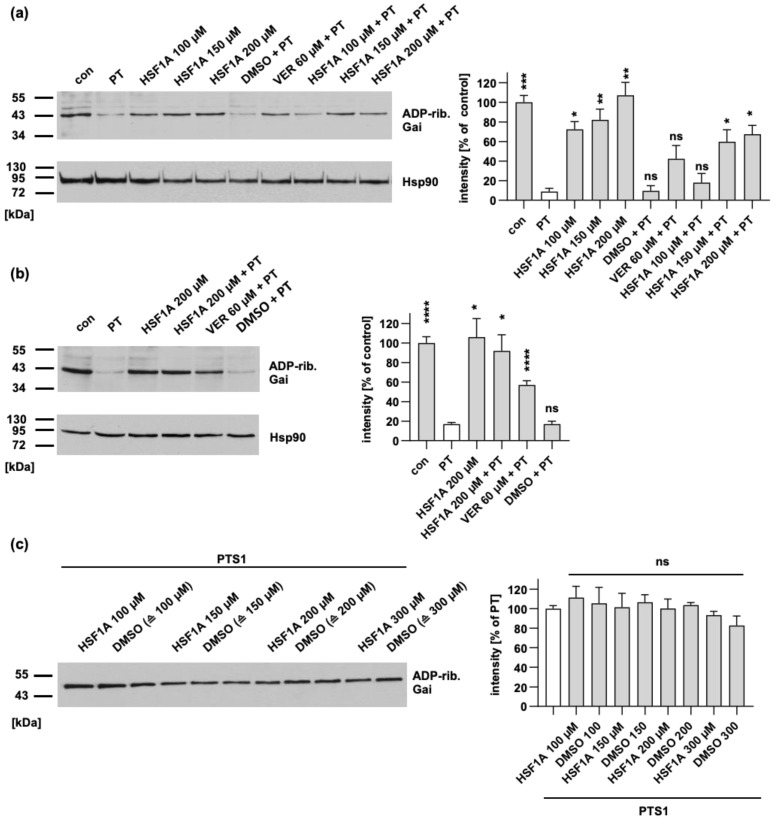
HSF1A leads to a reduction in the levels of ADP-ribosylated Gαi within cells, while it has no impact on the enzymatic activity of PTS1. (**a**) CHO cells were subjected to pre-incubation with either HSF1A or VER for 1 h at 37 °C. In comparison, untreated cells (con) serve as the control. For further control, cells were treated with the solvent of both inhibitors, DMSO, at concentrations corresponding to the 200 µM HSF1A. Following this, the cells were exposed to 10 ng/mL PT for 4 h, both with and without the respective inhibitors. The cellular lysates were then examined to determine the ADP-ribosylation status of Gαi through incubation with 100 nM PTS1 in the presence of biotin-NAD^+^. The presence of biotin-labeled (ADP-ribosylated) Gαi was detected, and the results were normalized to the loading control, confirmed by Hsp90 detection. The values displayed indicate the signal percentage relative to untreated cells, and are normalized to the respective loading control, with mean ± SEM values obtained from at least five values across five independent experiments. Significance is evaluated using mixed-effects analysis and Dunnett’s multiple comparisons test, with values compared to samples treated solely with PT (white bar). (**b**) A549 cells were pre-incubated with HSF1A, or DMSO (corresponding to 200 µM HSF1A) as the control, for 30 min at 37 °C. Similar to panel (**a**), 10 ng/mL PT was introduced to the cells for a 4 h period in the presence, or absence, of the inhibitors. The lysates were then analyzed to assess the ADP-ribosylation status of Gαi. Comparable protein loading was again confirmed by Hsp90 detection, and the results were expressed as the signal percentage relative to untreated cells, normalized to the Hsp90-based loading control. Mean ± SEM values from no less than five values across five independent experiments are provided, with significance determined through mixed-effects analysis and Dunnett’s multiple comparisons test, referring to samples treated only with PT (white bar). (**c**) Recombinant Gαi was pre-incubated with either HSF1A, or DMSO as the control, for 30 min at room temperature. For further control, recombinant Gαi was also incubated with buffer exclusively. Subsequently, 100 nM PTS1 and 10 μM biotin-labeled NAD^+^ were added, and incubation was carried out for 30 min at room temperature. The presence of biotin-labeled (ADP-ribosylated) Gαi was identified using streptavidin–peroxidase, and signal intensities were quantified via densitometry. The values are presented as a percentage relative to samples treated solely with PTS1, with mean ± SEM values from no less than four values across four independent experiments. Significance is determined through mixed-effects analysis and Dunnett’s multiple comparisons test, referring to samples treated only with PTS1 (white bar). **** *p* < 0.0001, *** *p* < 0.001, ** *p* < 0.01, * *p* < 0.05, ns = not significant.

Given that the uptake of PT into cells requires the activity of the chaperones Hsp90, Hsp70, Cyps, and FKBPs [20,50], we investigated whether the inhibitor of the chaperonin TRiC/CCT, HSF1A, also hinders the cell’s susceptibility to PT intoxication. In this pursuit, we focused on CHO cells (Figure 1a) due to their consistent and reliable ability to exhibit PT intoxication. Notably, these cells are also utilized to monitor residual PT activity during vaccine production [55].

To evaluate the effects, cells were exposed to PT both with and without HSF1A. For control, cells were treated with the established Hsp70 inhibitor VER [56]. Subsequently, the cells were lysed and then subjected to incubation with recombinant PTS1 in the presence of biotin-labeled NAD^+^. This enabled the biotin-labeling of Gαi that remained unmodified, i.e., not ADP-ribosylated, during the PT intoxication of the living cells. The results in Figure 1a demonstrate that the control samples exhibited strong signals of biotin-labeling, i.e., ADP-ribosylated Gαi, whereas weaker signals were observed in the samples of cells treated with PT. This shows that a significant proportion of Gαi in PT-treated cells had already undergone ADP-ribosylation, making it unsuitable for subsequent in vitro ADP-ribosylation. Remarkably, pretreatment of cells with HSF1A or VER before PT intoxication effectively diminished the ADP-ribosylation of Gαi in living cells, resulting in a stronger signal. This observation indicates that HSF1A offered protection against PT-induced cellular intoxication. It is noteworthy that treating cells with inhibitors alone had a negligible impact on the subsequent in vitro ADP-ribosylation of Gαi by PTS1, as depicted in Figure 1a.

In addition, A549 human lung epithelial cells (Figure 1b) were used, given their greater relevance to the disease’s pathology since whooping cough primarily affects the respiratory tract. Similar to CHO1 cells, HSF1A was shown to avert PT intoxication, i.e., we detected increased in vitro ADP-ribosylation of Gαi with recombinant PTS1 after the cells were co-incubated with HSF1A and PT (Figure 1b).

Moreover, we determined whether HSF1A could directly affect the enzymatic activity of PTS1. As shown in Figure 1c, we detected comparable levels of biotin-labeled recombinant Gαi in samples treated with different concentrations of HSF1A and recombinant PTS1 compared to samples treated solely with recombinant PTS1.

**Figure 2:**
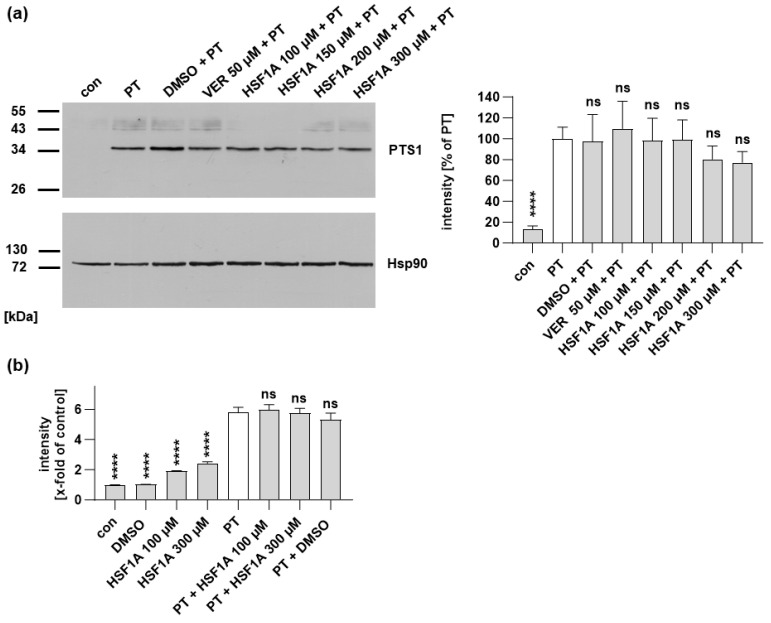
HSF1A does not inhibit the binding of PT to cells. (**a**) CHO cells were subjected to a 30 min pre-incubation with either HSF1A or VER, while DMSO was employed as a control, all carried out at 37 °C. Subsequently, cells were exposed to a total of 500 ng/mL PT at a temperature of 4 °C for a duration of 40 min. After thorough washing, the presence of bound PT was detected using Western blotting and a specific PTS1 antibody. Assurance of equivalent loading was validated through Hsp90 detection. The Western blot signals were quantified and then normalized both to the Hsp90 signals and to the samples treated solely with PT (mean ± SEM values), derived from a minimum of four values across four independent experiments. Statistical significance was assessed using mixed-effects analysis and Dunnett’s multiple comparisons test, with the values referring to samples treated exclusively with PT being represented by a white bar. (**b**) A suspension of CHO cells underwent a 30 min pre-incubation with HSF1A or DMSO at 37 °C. Subsequently, the cells were cooled on ice and then exposed to 500 ng/mL PT labeled with 488-dye for a duration of 15 min under ice-cold conditions. After undergoing two wash cycles, the attached PT was identified using flow cytometry. The values presented are indicated as x-fold relative to the control. The dataset comprises 10 values from five separate experiments. Statistical analysis involved a one-way ANOVA test, with the results compared to samples treated exclusively with PT. **** *p* < 0.0001, ns = not significant.

Another step of the intoxication process that might be influenced by HSF1A is the binding of PT to cells. To assess whether HSF1A influences PT binding on the cell surface, experiments were conducted at 4 °C, a temperature that facilitates PT binding but not internalization through endocytosis. The binding of PT to cells was then detected from cell lysates using a specific antibody against PTS1 in a Western blot analysis. Importantly, it was found that HSF1A did not hinder PT’s binding to cells, as shown in Figure 2a. As an alternative method, binding of PT to cells was assessed using flow cytometry. Figure 2b illustrates that HSF1A did not prevent the binding of PT to cells.

### 2.2. HSF1A Leads to Reduced Signal of PTS1 in Cells

**Figure 3:**
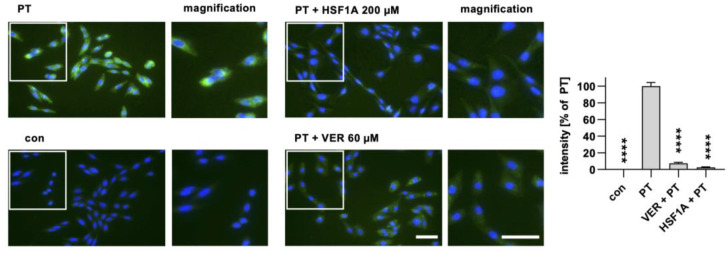
When cells are treated with HSF1A, a reduced amount of PTS1 signal is observed within cells. CHO cells were subjected to a 30 min pre-incubation with HSF1A or VER at 37 °C. For control, cells were left untreated. Following this, PT at a concentration of 100 ng/mL was introduced for a 4 h period. After washing, the cells underwent fixation and permeabilization, followed by staining of PTS1 through incubation with a specific primary antibody, succeeded by a secondary fluorescence-labeled antibody. Nuclei were stained using Hoechst dye, and images were captured in a random fashion using a Keyence fluorescence microscope. The PTS1 signal was quantified from uncropped images (40× objective) and values are shown as percent of PT-only treated cells (mean ± SEM, n = 30 (30 images from three independent experiments). An average of 41.73 ± 1.019 (mean ± SEM) cells were captured per image. Statistical analysis involved a mixed-effects analysis and Dunnett’s multiple comparisons test, with the results compared to samples treated exclusively with PT. **** *p* < 0.0001. Green = PTS1, blue = nucleus. White squares indicate the magnified areas. Scale bar = 50 µm.

After demonstrating that HSF1A does not interfere with PT binding to cells, nor with enzyme activity in vitro, we next investigated whether the trafficking of PTS1 into the cytosol is affected by HSF1A. Therefore, we employed a specific antibody targeting PTS1, which exhibits a preference for recognizing PTS1 when it is dissociated from the B-subunit pentamer, signifying mostly cytosolic PTS1 localization [51,52]. The fluorescence signals emanating from PTS1 were diminished in the samples treated with HSF1A or VER, in contrast to those treated solely with PT (Figure 3). These findings imply that HSF1A, similar to VER and other chaperone inhibitors [51,52,57], hampers the translocation of PTS1 into the cytosol of cells.

### 2.3. PTS1 Is Detected in Close Proximity with CCT5 in Cells

**Figure 4:**
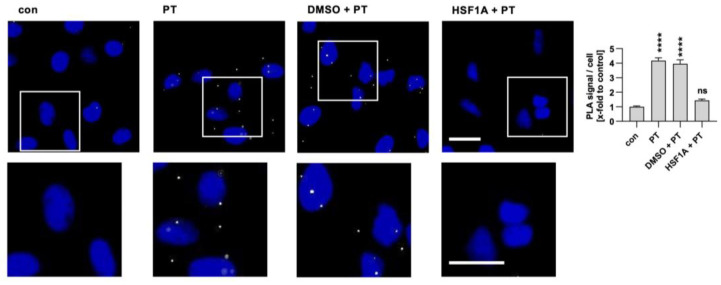
Interaction of TRiC/CCT subunit CCT5 with PTS1 in cells. A549 cells were pre-incubated with 200 µM HSF1A or the corresponding amount of its solvent, DMSO, for 30 min. Cells were challenged with 100 ng/mL PT for 4 h. Then, cells were carefully washed, fixed, and processed for a fluorescence-based proximity ligation assay (PLA) in accordance with the manufacturer’s guidelines. The cell nuclei were stained with Hoechst (blue). The resulting PLA signals (depicted in white) indicate instances of protein interaction between PTS1 and CCT5. White squares indicate the magnified areas below. The quantification of these signals per cell number is presented in the bar graph. The values are reported as mean ± SEM (n = 40 uncropped images captured in a random fashion using a Keyence fluorescence microscope 40× objective per condition from four independent experiments). An average of 37.09 ± 0.5213 (mean ± SEM) cells were captured per image. Significance levels tested using one-way ANOVA with Dunnett’s multiple comparisons test are indicated by asterisks (**** *p* ≤ 0.0001, ns denotes non-significant) and were tested against untreated control samples. Scale bar = 25 µm.

It has been demonstrated that *C. difficile* TcdB interacts with the subunits CCT4/5 of the TRiC/CCT chaperonin system [47]. Here, we investigated the interaction of PTS1 with the CCT5 subunit within cells using the proximity ligation assay (PLA). An amplified fluorescence signal is generated only when two specific antibodies come into close proximity within cells, which indicates that the two proteins most likely interact with each other. The interaction signals between PTS1 and CCT5 were significantly increased in cells exposed to PT compared to untreated control samples (Figure 4). Notably, the solvent for HSF1A, DMSO, did not influence this interaction. However, when cells were treated with HSF1A, the interaction between PTS1 and CCT5 was significantly diminished in comparison to cells treated solely with PT.

### 2.4. HSF1A Mitigates the Effect of PT on cAMP Signaling in Cells

Within the cytosol, the PTS1-mediated ADP-ribosylation disrupts the ability of Gαi to bind to GPCRs [37,58]. Consequently, the efficacy of agonist stimulation of inhibitory GPCRs is compromised, as Gαi becomes ineffective at efficiently inhibiting adenylate cyclase activity. To assess PT’s impact on cAMP signaling in living cells, the iGIST bioassay was employed [59]. This assay employs HEK293 cells that overexpress the Gαi-coupled GPCR somatostatin receptor 2 (SSTR2, inducible with octreotide) along with a luminescent cAMP probe. These cells are treated with forskolin to stimulate adenylate cyclase activity and with octreotide to activate SSTR2. The activation of SSTR2 results in decreased cAMP levels due to the Gαi-mediated inhibition of adenylate cyclase. PT has the ability to reverse this effect.

**Figure 5:**
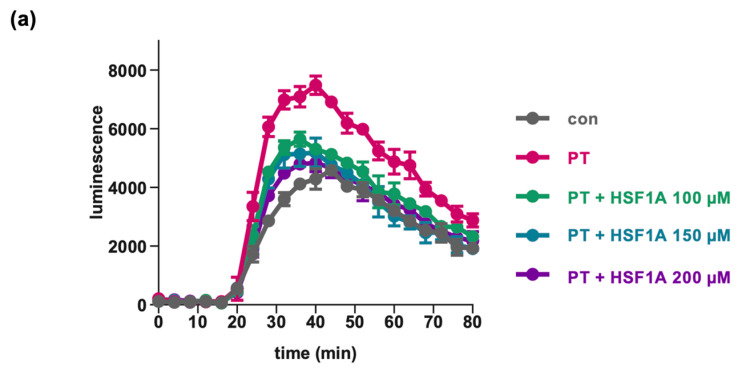

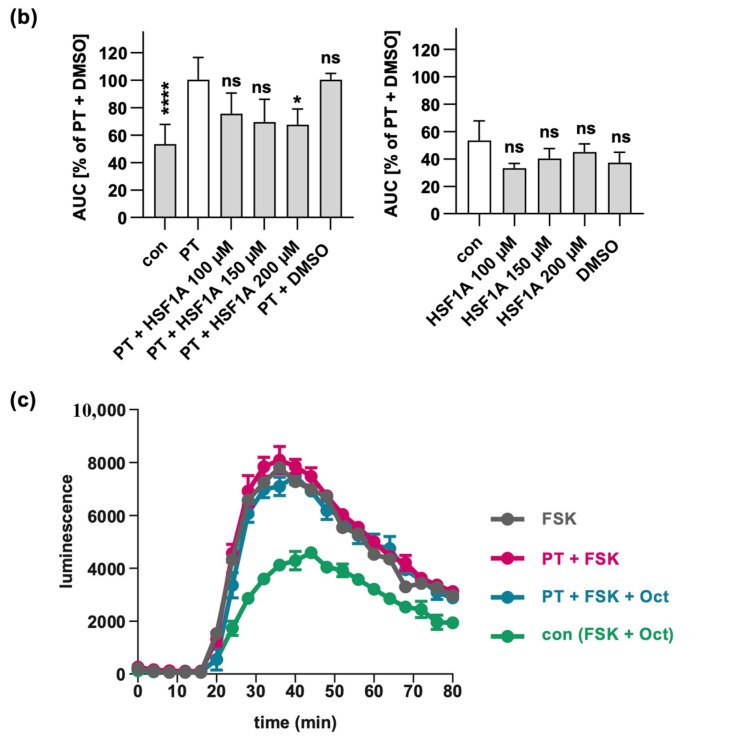
HSF1A mitigates the impact of PT on cAMP signaling. Cells were exposed to varying concentrations of HSF1A or DMSO (corresponding to 300 µM HSF1A) and 100 ng/mL of PT for a 5 h duration at 37 °C or were left untreated as a control. Subsequently, the inducing medium containing a luciferase substrate for the luminescent cAMP biosensor was introduced. A 15 min baseline measurement was taken, followed by the addition of forskolin (FSK), an adenylate cyclase activator, and octreotide acetate (Oct), an activator of SSTR2. Luminescence data were recorded over an 80 min period. (**a**) cAMP kinetic curves from a single representative experiment are shown. The values are presented as mean ± SD, derived from three samples within a single experiment. In panel (**b**), the bar graph illustrates the baseline-subtracted area under the curve (AUC) gathered from at least three separate experiments. The values are expressed as a percentage in relation to samples treated solely with PT + DMSO. The mean ± SEM values are provided, obtained from at least three values of five independent experiments. (**c**) For control, cells were treated with FSK only, or PT in combination with FSK, excluding Oct, to observe the maximal cAMP response. The values for control samples and samples treated with PT, FSK, and Oct mirror those in panel (**b**). The statistical analysis involved a mixed-effects assessment along with Dunnett’s multiple comparisons test. The values presented correspond to samples treated exclusively with PT (left bar graph, white bar) or the control (right bar graph, white bar). **** *p* < 0.0001, * *p* < 0.05, ns = not significant.

Exposure of cells to HSF1A led to a decrease in cAMP signal compared to cells treated solely with PT (Figure 5). The inhibiting effect was significant if HSF1A was applied at the highest concentration of 200 µM. The inhibitor alone had no significant effect on the intracellular cAMP levels measured in the bioassay (Figure 5b, right graph). The peak cAMP levels were reached when cells were solely treated with forskolin, or when cells were exposed to PT in conjunction with forskolin and octreotide (Figure 5c). These findings suggest that HSF1A is capable of mitigating PT-induced alterations in host cell cAMP signaling.

Taken together, our findings demonstrate that in PT-treated cells, HSF1A effectively decreases ADP-ribosylated Gαi levels without adversely affecting enzyme activity in vitro or the cellular binding of PT. Furthermore, HSF1A leads to a reduction in the PTS1 signal within cells and a reduction of the interaction between PTS1 and CCT5 in cells. HSF1A also mitigates the impact of PT on cAMP signaling. These findings indicate a crucial role of TRiC/CCT in the uptake of PT into cells.

## 3. Discussion

The uptake and translocation of PT into host cells are intricate processes important for the pathogenicity of *Bordetella pertussis*, the causative agent of whooping cough. Understanding the molecular mechanisms underlying these processes is fundamental for developing therapeutic strategies against whooping cough and related diseases. Here, we delve into the role of host cell chaperones, particularly the chaperonin TRiC/CCT, in facilitating the efficient uptake and translocation of PTS1 into the host cell cytosol.

**Figure 6:**
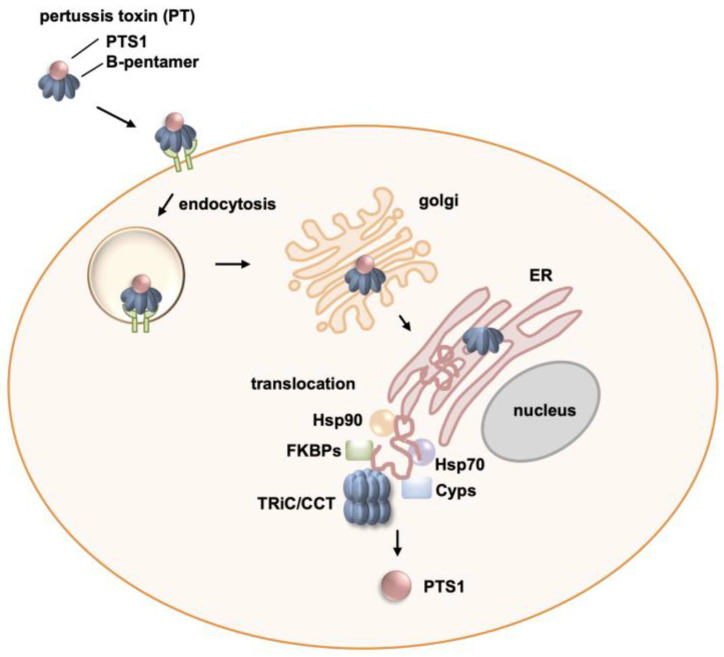
Schematics of the role of host cell chaperones in pertussis toxin activity. Pertussis toxin (PT) consists of the enzyme subunit PTS1 and the B-subunit pentamer, which facilitates cell binding. The cell binding is followed by endocytosis and retrograde transport of the toxin through the Golgi to the endoplasmic reticulum (ER). In the ER, PTS1 is released from the B-pentamer, unfolded, and transported to the cytosol by the ER-associated degradation pathway. This translocation and subsequent refolding of PTS1 is supported by several chaperones, Hsp90, Hsp70, cyclophilins (Cyps), and FK506 binding proteins (FKBPs), as well as the chaperonin TRiC/CCT complex.

Uptake of PT into cells involves receptor binding, endocytosis, retrograde transport through the Golgi to the ER, and transport of PTS1 into the cytosol utilizing the ERAD pathway [60]. Crucial for reaching the cytosol is the unfolding of PTS1, which occurs under physiological temperatures, i.e., 37 °C [33,34]. TRiC/CCT was shown to play a pivotal role in this process, as demonstrated by the reduced detection of PTS1 in cells upon inhibition of TRiC/CCT with HSF1A. Moreover, the subunit CCT5 of the TRiC/CCT was found in close proximity with PTS1 in cells, and the proximity/interaction signal was reduced by HSF1A. Protection from PT intoxication of cells by HSF1A was also demonstrated by the reduced ADP-ribosylation of PT’s substrate Gαi, and the diminished effects of PT on cAMP signaling if cells were treated with HSF1A. These effects occurred without affecting receptor binding of PT or enzyme activity of PTS1 in vitro, indicating that HSF1A interferes with the uptake/translocation and/or refolding of PTS1 in the cytosol. Figure 6 depicts the current model detailing the involvement of TRiC/CCT alongside other chaperones in facilitating cellular uptake, particularly in the translocation process of PTS1 into the cytosol.

Interestingly, a requirement of TRiC/CCT was also shown for *C. difficile* toxins TcdB and TcdA, as well as other members of the glucosylating toxin family [47]. These toxins exert their harmful effects within host cells by adding glucose molecules to intracellular target proteins. Following their binding to receptors and subsequent endocytosis, these toxins enter the host cell cytosol, where they modify specific target proteins, notably Rho proteins [61]. It was shown that the subunits CCT4/5 of the TRiC/CCT chaperonin system directly interact with these toxins, facilitating the refolding and restoration of the toxins’ glucosyltransferase activities, particularly following exposure to heat treatment. Here too, the cytotoxic effects of TcdA and TcdB were effectively blocked by HSF1A [47].

Recently, the approved antiemetic drug domperidone was discovered as a novel inhibitor of Hsp70 activity [62]. In a previous study, we demonstrated that domperidone protects cells from intoxication with PT since Hsp70 activity is also needed for the uptake of PT into cells [57]. In that study, we also showed that PTS1 activity in vitro is reduced at 37 °C compared to at room temperature and that in the presence of Hsp70 or Hsp90 enzyme activity is stronger at both temperatures, suggesting that Hsp70 and Hsp90 are required to restore enzyme activity [57]. A comparable mechanism of action was shown for the enzyme subunit of TcdB in the presence of TRiC/CCT components [47]. Therefore, it can be assumed that TRiC/CCT components restore the enzyme activity of PTS1 after translocation, as well. Moreover, it was shown for PTS1, but also for the related cholera toxin, that chaperone interaction not only occurs during translocation but that the toxin-chaperone complex was detected in cells, or in vitro, for up to 48 h after intoxication [52,63,64,65]. This might indicate that PTS1 need the chaperones permanently to ensure proper enzyme activity and possibly to protect the toxin enzyme subunit from degradation.

It was also shown that HSF1A had no effect on the ADP-ribosylating toxins C2 toxin and CDT [47]. However, it was demonstrated previously that C2 toxin, CDT, and other toxins, but not TcdA and TcdB, depend on the activity of the chaperones Hsp90, Cyps, and FKBPs [47,66,67,68,69,70,71,72]. In the uptake and/or refolding of PT, these requirements are all combined: PT needs Hsp90, Hsp70, Cyps, and FKBPs, as well as the activity of TRiC/CCT, for efficient intoxication of cells (Figure 6) [51,52,53,54]. The role of chaperones during the uptake of bacterial AB-type toxins has been the subject of several investigations in the past and was reviewed recently [50]. So far, it is not clear whether the different chaperones act in a coordinated manner or independently. For Hsp70, Hsp90, Cyps, and FKBPs, a coordinated action was described during the folding and activation of steroid hormone receptors [73,74,75]. It was previously observed that combinations of chaperone inhibitors increased the inhibitory effect on intoxication, for example, with C2 toxin [67,68,76,77]. However, if the chaperones indeed function in a coordinated manner, the inhibition of either partner should suffice to disrupt the entire process. It became apparent that there are several common requirements, e.g., the majority of investigated toxins require Hsp90 activity, but also several differences were discovered, e.g., most investigated ADP-ribosylating toxins depend on Hsp90, Cyps, FKBPs, and Hsp70, but botulinum neurotoxin only requires Hsp90 and not Cyps or Hsp70 [78,79]. A common pattern or distinct characteristic determining which chaperones are needed has not been established thus far. The finding that PT requires TRiC/CCT, as well as Hsp90, Hsp70, Cyps, and FKBPs, provides a further piece to the puzzle of understanding the underlying common characteristics.

In conclusion, our study demonstrates that the entry of PT into host cells relies on the activity of the TRiC/CCT chaperonin complex, as confirmed by the successful inhibition of TRiC/CCT using the specific inhibitor HSF1A. This discovery sheds light on the crucial role of TRiC/CCT in bacterial pathogenesis and opens up new avenues for potential therapeutic interventions. By targeting the TRiC/CCT complex, we may develop more effective treatments for pertussis and other related infections, highlighting the broader significance of chaperonin complexes in cellular processes and their potential as targets for future antimicrobial strategies.

## 4. Materials and Methods

### 4.1. Protein Expression and Purification

Recombinant proteins were expressed and purified following the previously outlined methods: PTS1 and Gαi [80]. His-tagged PTS1 and Gαi were expressed in BL21(DE3) and purified using HisTrap HP columns followed by size exclusion chromatography.

### 4.2. Cell Culture

Chinese hamster ovary cells strain K1 (CHO-K1) were procured from DSMZ (Leibniz Institute DSMZ-German Collection of Microorganisms and Cell Cultures GmbH), Braunschweig, Germany) and cultured in a mixture of DMEM and HAM’s F12 supplemented with 5% heat-inactivated fetal calf serum, 1 mM sodium pyruvate, and penicillin–streptomycin (1:100). The cells were maintained at a temperature of 37 °C with 5% CO_2_. A549 human lung adenocarcinoma cells were obtained from ATCC and cultivated at 37 °C with 5% CO_2_ in DMEM supplemented with 10% FCS, 1 mM sodium pyruvate, 0.1 mM non-essential amino acids, 100 U/mL of penicillin, and 100 μg/mL of streptomycin. The cells were trypsinized and transferred to a 10 cm culture dish every two to three days for a maximum of 25 cycles.

### 4.3. Sequential ADP-Ribosylation of Gαi in Lysates from Toxin-treated Cells

Cells were pre-incubated at 37 °C with specific inhibitors and subsequently treated with PT (10 ng/mL, Merck Sigma, Darmstadt, Germany) for designated incubation periods. After treatment, the cells were washed three times with PBS and then frozen at −20 °C overnight for cell lysis. The frozen and lysed cells were scraped off in 30 μL of ADP-ribosylation buffer, containing 0.1 mM Tris-HCl (pH 7.6), 20 mM DTT, and 0.1 μM ATP, supplemented with complete protease inhibitor (Roche, Basel, Switzerland). Next, the lysed cells were incubated with 100 nM PTS1 and biotin-labeled NAD^+^ (8.3 μM; R&D Systems, Minneapolis, MA, USA) at room temperature for 40 min to facilitate the in vitro ADP-ribosylation of Gαi, which had not undergone ADP-ribosylation during the previous intoxication step.

Following the incubation, samples were subjected to SDS–PAGE, blotted, and detection of biotin-labeled (i.e., ADP-ribosylated) Gαi was achieved using streptavidin–peroxidase (Strep-POD, Sigma-Aldrich, Merck, St. Louis, MO, USA) with the enhanced chemiluminescence (ECL) system. To ensure equal protein amounts, Ponceau S staining and Hsp90 detection with a specific antibody (Santa Cruz, Dallas, TX, USA) were performed. Densitometric quantification of Western blot signals was carried out using the Image J histogram tool (v1.53k, National Institute of Health, Bethesda, MD, USA), and the values were normalized based on the amount of loaded protein.

### 4.4. In Vitro Enzyme Activity

Recombinant Gαi (0.8416 μM) underwent a 30 min incubation at room temperature with specific inhibitors. As a control, Gαi was also incubated with DMSO, which served as the solvent for the inhibitors. The final concentration of DMSO was compared to the highest DMSO concentration utilized in the inhibitor experiments. Following the 30 min incubation, 100 nM PTS1 and 10 μM biotin-labeled NAD^+^ were introduced and allowed to incubate for an additional 30 min at room temperature. The samples were then subjected to SDS–PAGE and transferred onto a nitrocellulose membrane. Streptavidin–peroxidase was used to detect biotin-labeled (i.e., ADP-ribosylated) Gαi, and the intensity of the signals was quantified with densitometry using Image J. To confirm comparable protein loading, Ponceau S staining was performed.

### 4.5. Binding of PT to Cells by Western Blot Analysis and Flow Cytometry

To assess the impact of inhibitors on the binding of PT, cells were first treated with the respective inhibitors for 30 min at 37 °C, followed by incubation on ice for 15 min. PT was then introduced and allowed to incubate for 40 min on ice. Subsequently, the cells were washed twice with PBS, and Laemmli buffer containing DTT was added to each well. The samples were subjected to analysis using SDS–PAGE and Western blotting, with the specific antibody (Santa Cruz, #sc-57639 (63.1G9)) employed to detect PTS1. The intensity of the signal was quantified through densitometry using Image J, and the verification of comparable loading was performed by staining for Ponceau S and Hsp90.

For flow cytometry analysis, cells were released from culture dishes by employing 25 mM EDTA in PBS and then suspended in a serum-free medium. The cell suspension (1 × 10^5^ cells in 0.2 mL per sample) was subjected to a 1 h incubation with respective inhibitors at 37 °C. Then, 488-labeled PT was added for 15 min on ice. This ice-based incubation facilitated PT binding to the cell surface without internalization. To eliminate any unbound 488-labeled PT, cells were subjected to two washes via centrifugation. Cell fluorescence was quantified using a BD FACS Celesta™ flow cytometer (Becton, Dickinson and Company, Franklin Lakes, NJ, USA) along with BD FACSDivaTM software 8.0.1.1. DyLight488 excitation was executed using a blue laser (488 nm), and emitted fluorescence was captured using a 530 nm (530/30) bandpass filter. Data analysis of gated cell populations was executed using Flowing Software v2.5.1. (Turku Centre of Biotechnology, Turku, Finland). DyLight488 NHS Ester (Thermo Fisher Scientific, Rockford, IL, USA) was employed to label PT in accordance with the manufacturer’s instructions, and excessive dye was eliminated via ZebaTM Spin Desalting Columns (7K MWCO, Thermo Fisher Scientific, Waltham, MA, USA).

### 4.6. Immunolabeling and Fluorescence Microscopy

CHO cells were plated in ibidi 8-well µ-plates. The medium in the wells was replaced with either and inhibitor-containing medium or a medium without an inhibitor, serving as the control. The cells were then incubated for 30 min at 37 °C. Following the incubation, PT was added to the wells and allowed to incubate for 4 h. Subsequently, the cells were washed three times with cold PBS and fixed with 4% PFA for 20 min at room temperature (RT). After another round of three cold PBS washes, the cells were permeabilized with Triton X-100 (0.4% in PBS) for 5 min at RT and washed three more times with PBS. To quench autofluorescence, the cells were treated with 100 nM glycine in PBS for 2 min at RT and washed again with PBS.

Next, the cells were blocked with 10% normal goat serum (Jackson ImmunoResearch, Philadelphia, PA, USA) and probed with an anti-PTS1 antibody (diluted 1:200 in the blocking solution) for 1 h at 37 °C. After four washes with PBST, the cells were probed with a fluorescence-labeled secondary antibody, anti-mouse488 (diluted 1:1000 in the blocking solution; Invitrogen, Waltham, MA, USA), and Hoechst stained (diluted 1:10,000 in PBST) for 5 min. The cells were then washed five times before imaging, which was performed using a Keyence BZ-X810 fluorescence microscope (Osaka, Japan) with a Plan Apochromat 40× objective and BZ-X filters for DAPI (OP-87762) and GFP (OP-87763). PTS1 signals were quantified from uncropped images using Image J histogram analysis.

### 4.7. Proximity Ligation Assay (PLA) for Detection of Protein Interaction in Cells

Cells were cultured in 18-well μ-slides (ibidi GmbH, Gräfelfing, Germany), pre-incubated with respective inhibitors, and exposed to PT for a 4 h incubation at 37 °C. Subsequently, the cells underwent a series of steps: they were rinsed with PBS, fixed with 4% PFA, permeabilized, and blocked with a solution containing 10% NGS and 1% BSA in PBS-T. Following this, the cells were treated with primary antibodies, namely rabbit anti-PT (Abcam, Cambridge, UK) and mouse anti-CCT5 (Abcam) or mouse anti-α-defensin-5 (Abcam). This antibody incubation took place for 1 h at 37 °C. PLA was executed in accordance with the manufacturer’s guidelines, utilizing Duolink’s PLA technology (Sigma-Aldrich Merck, Darmstadt, Germany). PLA secondary antibodies were employed, with anti-rabbit antibodies for detecting PTS1 and anti-mouse antibodies for detecting CCT5. These secondary antibodies featured specific oligonucleotide sequences. When these oligonucleotides came into close proximity they could assemble into a ring structure. The addition of ligase and polymerase facilitated rolling-circle amplification. Samples were probed with oligonucleotides labeled for fluorescence, which were complementary to the amplification product, thus enabling the detection of protein interactions. The resulting PLA signals were quantified by counting them from fluorescence images using the Image J find maxima tool.

### 4.8. iGIST Bioassay

The iGIST bioassay [27] employs HEK293 cells that have been genetically modified to express the Gαi-coupled somatostatin receptor 2 (SSTR2) GPCR along with a luminescent cAMP probe, called GloSensor-22F, from Promega (Madison, WI, USA). These cells were seeded in 96-well plates with white walls and a translucent bottom (View-Plate 96, Perkin Elmer, Waltham, MA, USA). The cells were first treated with the inhibitors for 1 h. Then, PT (100 ng/mL) or a matched buffer, SolC (composed of 50% glycerol, 50 mM Tris, 10 mM glycine, 0.5 M NaCl, pH 7.5), was added for 5 h. After removing the medium, the cells were treated with an inducing medium consisting of 2% GloSensor reagent (Promega) and 400 μM of the phosphodiesterase inhibitor IBMX from Sigma, in a mixture of DMEM/F12 medium and CO_2_-independent medium (with a ratio of 4 parts DMEM/F12 per 5 parts CO_2_-independent medium). The medium was further supplemented with 0.1% (*w*/*v*) bovine serum albumin. The plate was then equilibrated for 45 min at room temperature in the dark, and the baseline luminescence was recorded for 15 min using the Orion microplate luminometer from Berthold Detection Systems (Oak Ridge, TN, USA). Afterward, the cells were stimulated by adding forskolin (10 μM, Merck Sigma) to activate adenylate cyclase, and octreotide acetate (20 nM, Bachem, Bubendorf, Switzerland) to activate the Gαi-coupled SSTR2 GPCR, both in 25 mM HEPES buffer (pH 7.4). Luminescence, which reflects cAMP levels in the cells, was measured for an additional 60 min. To quantify the kinetic curves, the area under the curve (AUC) was calculated after subtracting the baseline using the GraphPad Prism software.

### 4.9. Reproducibility of Experiments and Statistics

Each experiment was conducted independently on a minimum of three occasions. The number of replicates for each experiment is specified in the corresponding figures. The figures present representative results. For display purposes only, Western blots were cropped. Statistical analysis was performed as described in the figure legends, using the GraphPad Prism software, with significance levels denoted as follows: **** *p* < 0.0001, *** *p* < 0.001, ** *p* < 0.01, * *p* < 0.05, and ns = not significant (*p* > 0.05).

## Data Availability

The datasets generated during and/or analyzed during the current study are available from the corresponding author upon reasonable request.
